# scPASU: A computational protocol for quantifying polyadenylation site usage and alternative polyadenylation from 3′ scRNA-seq data

**DOI:** 10.1016/j.xpro.2026.104544

**Published:** 2026-05-04

**Authors:** Alexandra Krylova, Ninh B. Le, Angela H. Ting

**Affiliations:** 1 Department of Epigenetics and Molecular Carcinogenesis, The University of Texas MD Anderson Cancer Center, Houston, TX 77054, USA

**Keywords:** Bioinformatics, Sequence analysis, RNAseq

## Abstract

3′ single-cell RNA sequencing (scRNA-seq) captures polyadenylation (poly(A)) sites, enabling quantification of site usage per gene and cell. Here, we present scPASU (single-cell poly(A) site usage), a Snakemake workflow for quantifying poly(A) site usage and alternative polyadenylation from 3′ scRNA-seq data. We describe steps for building a poly(A) site reference, generating a site-by-cell matrix per sample, and testing alternative polyadenylation (APA) between cell groups. This protocol is configurable for organism- and sample-specific parameters and supports discovery of poly(A) sites.

For complete details on the use and execution of this protocol, please refer to Le et al.^1^

## Highlights

• Workflow to generate a novel poly(A) isoform reference from scRNA-seq data
• Steps for creating a cell-by-poly(A) peak matrix from Cell Ranger BAM files
• Guidance on performing alternative polyadenylation testing between different cell types

## Abstract (editor highlights)

Publisher’s note: Undertaking any experimental protocol requires adherence to local institutional guidelines for laboratory safety and ethics.

## Graphical abstract

**Figure:**
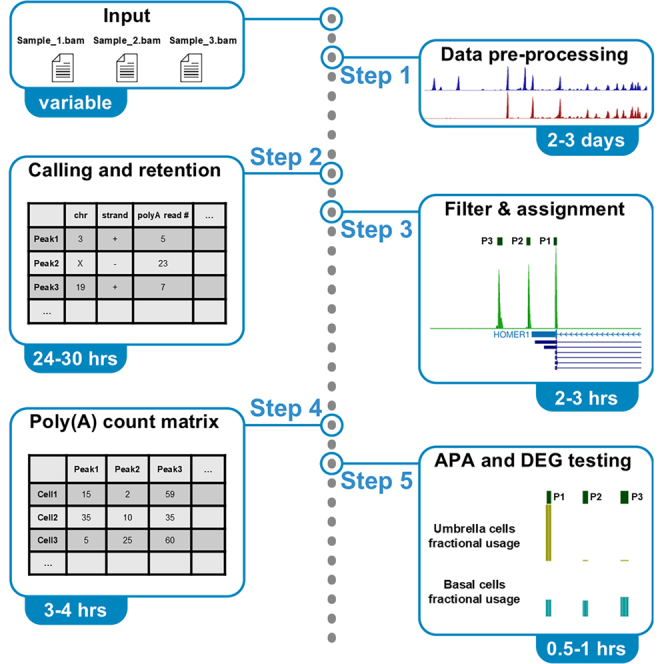

## Before you begin

Alternative polyadenylation (APA) is a pervasive yet underexplored mechanism that expands transcriptome diversity in a cell- and tissue-specific manner. By generating mRNAs with distinct 3′ untranslated regions, APA fine-tunes expression through effects on stability, localization, and translation efficiency,^2^^,^^3^^,^^4^^,^^5^^,^^6^^,^^7^^,^^8^^,^^9^ and, in some cases, alters coding potential, as in the immunoglobulin switch from membrane-bound to secreted forms in B cells.^10^ Despite its broad impact, APA remains incompletely characterized in complex tissues because conventional bulk 3′-end sequencing averages signals across cells and obscures cell type-specific APA dynamics.^11^ Resolving APA isoforms at single-cell resolution is therefore needed.

scPASU (single-cell Poly(A) Site Usage) is a Snakemake-based workflow that extracts APA information from standard 3′ scRNA-seq data. Conceptually, scPASU builds a dataset-specific polyadenylation (poly(A)) site reference from junction evidence, generates per-sample site-by-cell count matrices using that reference, and tests multi-site genes for differences in site usage between specified cell groups. The protocol below describes the steps for using scPASU to analyze APA in human urothelial cell differentiation in the ureter.^1^ The urothelium spans from basal progenitors to terminally differentiated umbrella cells. Using scPASU, we mapped poly(A) isoforms and identified hundreds of differentiation-associated APA events among basal, intermediate, and umbrella cells. Most APA genes were not differentially expressed by total mRNA abundance, revealing regulatory dynamics that conventional analyses miss.

### Innovation

This protocol introduces scPASU, a Snakemake-based workflow that quantifies APA from standard 3′ scRNA-seq without specialized library preparation. The advance lies in constructing a dataset-specific poly(A) site reference directly from input BAM files, which enables discovery of novel poly(A) sites rather than relying on fixed annotations. scPASU applies transparent, user-tunable criteria for read deduplication, removal of genomic-A-primed artifacts, and peak retention; it also rescues low-coverage sites by combining junction-based evidence with peak calling. Poly(A) processing regions are inferred, and sites are assigned and ordered within transcription units for consistent, interpretable annotation.

The workflow is engineered for usability and reproducibility. A Snakemake wrapper installs required packages, manages dependencies, and executes steps in the correct order, so researchers with limited computational experience can run the pipeline end-to-end. It runs in parallel with routine scRNA-seq analyses, scales from a workstation to high-performance computing (HPC) systems, and is easily configurable for study-specific parameters. For statistical testing, scPASU adapts a DEXSeq-based framework to single-cell data by generating pseudo-replicates and enforcing explicit effect-size and usage thresholds, yielding robust detection of APA differences between defined cell groups. Together, these features provide an interpretable and generalizable path to single-cell APA analysis.

### Package installation and pipeline setup

**Timing: 30 min**

1. Install Snakemake as detailed here: https://snakemake.readthedocs.io/en/v7.32.0/getting_started/installation.html.

***Note:*** This pipeline has been tested with Snakemake version 7.32.4.

2. Install additional tools required to run the pipeline.
  a. Install Cell Ranger v.7.1.0 or higher as detailed here: https://www.10xgenomics.com/support/software/cell-ranger/latest/tutorials/cr-tutorial-in.
  b. Install the polyAfilter directory and note the location of the directory:
    >git clone https://github.com/MarekSvob/polyAfilter.git
  c. Install goldmine in R version 4.3.3 and note the location of the library:
    >if(!require(devtools)){
    > install.packages(“devtools”) # If not already installed
    >}
    >devtools::install_github(“jeffbhasin/goldmine”)
3. Set up the scPASU workflow.
  a. Clone scPASU repository.
    >git clone https://github.com/AngelaTingLab/scPASU_snakemake_version.git
  b. Move the scPASU_snakemake_version directory to where the pipeline is to be executed and outputs stored.
    ***Note:*** It is recommended to run the pipeline on an HPC for datasets larger than 25 GB.
  c. Navigate to the workflow directory under the scPASU_snakemake_version directory to run Snakemake commands.

### Running Cell Ranger alignment

**Timing: Variable depending on size of the dataset**

4. Run *cellranger count* on each sample.

>cellranger count --id={sample_name} --fastqs={fastq_path} --sample={sample_name} --transcriptome={transcriptome_path}

**CRITICAL:** If using cellranger v. 8.0 or higher, set --create-bam=true.

### Formatting metadata and barcode files

**Timing: 30 min**

5. If performing APA and differential gene expression (DEG) testing in Module 5, prepare the metadata and barcode files:
  a. Create a CSV metadata file, where the first column must be the cell barcodes for all cells and there must be an additional column containing cell group identities. Any additional columns will be ignored (Figure 1).

**Figure 1:**
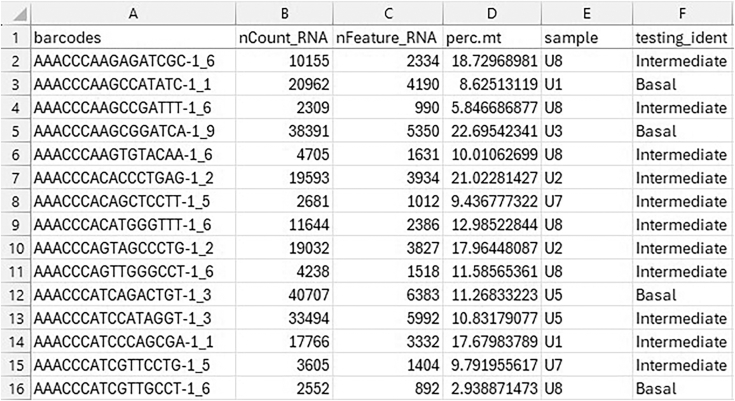
Expected format of the metadata file The first column must contain the cell barcodes, and the file must include a column specifying the testing groups (referred to here as “test_ident”). Additional columns, such as sample identifiers, RNA counts, or other metadata, may also be included.
  b. Move the CSV file to the “scPASU_snakemake_version/workflow/data” directory.
6. If only running scPASU on a subset of cells in the dataset, prepare sample barcode files:
  a. Create tsv files for each sample containing cell barcodes to keep where each row is one cell barcode without quotation marks or file header.
  b. Name the files as “[sample_name]_subset_barcodes.tsv”.
  c. Place tsv files in the “scPASU_snakemake_version/workflow/data/subset_bams” directory.

### Setting up the configuration file

**Timing: 10–20 min**

7. Navigate to the “scPASU_snakemake_version/config” directory.
8. Open the “config.yaml” file in a text editor and update the following variables to match your dataset and analysis:
  a. *compartment*: Prefix used for intermediate and output file names. Set this to the cell group being analyzed (e.g., “fibroblasts”).
  b. *genomename*: Genome reference to use. The pipeline currently supports GRCh38 for human (set parameter to “hg38”) and GRCm38 for mouse (set parameter to “mm10”).
  c. *chrs*: For human: “chr1,chr2,chr3,chr4,chr5,chr6,chr7,chr8,chr9,chr10,chr11,chr12,chr13,chr14,chr15,chr16,chr17,chr18,chr19,chr20,chr21,chr22,chrX, chrY” and for mouse: “chr1,chr2,chr3,chr4,chr5,chr6,chr7,chr8,chr9,chr10,chr11,chr12,chr13,chr14,chr15,chr16,chr17,chr18,chr19,chrX,chrY”
  d. *samples*: List of sample names to analyze, exactly matching the sample names in the Cell Ranger alignment output. Format as [“Sample_1”, “Sample_2”, “Sample_3”, …]
  e. *subset*: Set to “true” if analyzing a specific list of cell barcodes (rather than all barcodes in the Cell Ranger BAM files). If “true”, provide the barcode file paths in the barcodepath parameter below. Otherwise, set to “false” to analyze all barcodes.
  f. *PAS_filtering*: Set to “true” to perform PAS filtering or “false” to skip this step. See the Module 3 section for an explanation of this filtering step.
  g. *group_one* and *group_two*: Names of the two cell groups to compare during APA and DEG testing, matching the group labels in your metadata (e.g., group_one: “Umbrella”, group_two: “Basal”).
  h. *meta_testing_column*: Name of the metadata column that contains the cell group labels specified above (e.g., “cell_type”).
9. Continue in the same “config.yaml” file and update the following path variables to match the locations of files, tools, and directories on your system:
  a. *work_dir*: Absolute path to the scPASU workflow directory. Format: “/path/to/scPASU/workflow/”.
  b. *cellrangeroutputpath*: Absolute path to the directory containing all Cell Ranger alignment outputs. Format: “/path/to/cellranger/alignments”.
  c. *cellrangerrefpath*: Absolute path to the genome reference used for Cell Ranger alignment. Format: “/path/to/cellranger/reference/”.
  d. *barcodepath*: Relative path from the workflow directory to the folder containing per-sample tsv files listing barcodes of interest. If subset is “false”, leave this parameter as empty string: “”. Otherwise, set to directory such as “data/subset_bams”.
  e. *seurat_obj_file*: Relative path from the workflow directory to a Seurat object containing the cells of interest. This object is optional and only used during peak matrix merging in module 5 to ensure barcode formatting matches the object. Leave as “” if not used; otherwise, format as “data/seurat_object_name.rds”.
  f. *metadata_file*: Relative path from the workflow directory to a CSV file containing metadata for the cells of interest. Format: “data/metadata_file_name.csv”.
  g. *polyAfilterpath*: Absolute path to the polyAfilter package directory cloned from GitHub as described in the Package installation and pipeline set up section. Format: “/path/to/package/polyAfilter/”.
  h. *cellrangerpath*: Absolute path to the installed Cell Ranger software. Format: “/path/to/package/cellranger/version_number/”.
  i. *goldminepath*: Absolute path to the directory containing the goldmine R as described in the Package installation and pipeline set up section. Format: “/path/to/R/package/library”. Note that here, the “library” directory should contain the R package directory named “goldmine”.

## Key resources table

**Table:** 

REAGENT or RESOURCE	SOURCE	IDENTIFIER
**Software and algorithms**		

R version 4.3.3	CRAN	https://cran.r-project.org/
Python version 3.10	RRID: SCR_008394	https://www.python.org
apeglm version 1.24.0	Zhu *et al.*^12^	https://bioconductor.org/packages/release/bioc/html/apeglm.html
argparser version 0.7.2	CRAN	https://cran.r-project.org/web/packages/argparse/index.html
Bioperl version 1.7.8	Stajich *et al.*^13^	https://metacpan.org/pod/BioPerl
Biostrings version 2.70.1	Bioconductor	https://bioconductor.org/packages/release/bioc/html/Biostrings.html
BSgenome.Hsapiens.UCSC.hg38	Bioconductor	https://bioconductor.posit.co/packages/3.20/data/annotation/html/BSgenome.Hsapiens.UCSC.hg38.html
cluster version 2.1.8	CRAN	https://cran.r-project.org/web/packages/cluster/index.html
data.table version 1.15.2	CRAN	https://cran.r-project.org/web/packages/cluster/index.html
DESeq2 version 1.42.0	Love *et al.*^14^	https://bioconductor.org/packages/release/bioc/html/DESeq2.html
DEXSeq version 1.48.0	Anders *et al.*^15^	https://bioconductor.org/packages/release/bioc/html/DEXSeq.html
dplyr version 1.1.4	CRAN	https://bioconductor.org/packages/release/bioc/html/DEXSeq.html
edgeR version 4.0.16	Chen *et al.*^16^	https://bioconductor.org/packages/release/bioc/html/edgeR.html
GenomicRanges version 1.54.1	Lawrence *et al.*^17^	https://bioconductor.org/packages/release/bioc/html/GenomicRanges.html
ggplot2 version 3.5.1	CRAN	https://cran.r-project.org/web/packages/ggplot2/index.html
goldmine version 1.0.0	Bhasin *et al.*^18^	https://github.com/jeffbhasin/goldmine/
gtools version 3.9.5	CRAN	https://cran.r-project.org/web/packages/gtools/index.html
Igraphversion 2.0.3	CRAN	https://cran.r-project.org/web/packages/igraph/index.html
magrittr version 2.0.3	CRAN	https://cran.r-project.org/web/packages/magrittr/index.html
parallelly version 1.45.1	CRAN	https://cran.r-project.org/web/packages/parallelly/index.html
pheatmap version 1.0.12	CRAN	https://cran.r-project.org/web/packages/pheatmap/index.html
polyAfilter	Svoboda *et al.*^19^	https://github.com/MarekSvob/polyAfilter
Readr version 2.1.5	CRAN	https://cran.r-project.org/web/packages/readr/index.html
reshape version 0.8.9	CRAN	https://cran.r-project.org/web/packages/reshape/index.html
RcolorBrewer version 1.1	CRAN	https://cran.r-project.org/web/packages/RColorBrewer/index.html
Rsubread version 2.16.1	Liao *et al.*^20^	https://bioconductor.org/packages/release/bioc/html/Rsubread.html
Rtracklayer version 1.62.0	Lawrence *et al.*^21^	https://www.bioconductor.org/packages/release/bioc/html/rtracklayer.html
samToPolyA.pl	GitHub	https://github.com/julienlag/samToPolyA
Seurat version 5.1.0	Satija *et al.*^22^	https://cran.r-project.org/web/packages/Seurat/index.html
stringr version 1.5.1	CRAN	https://cran.r-project.org/web/packages/stringr/index.html
tibble version 3.2.1	CRAN	https://cran.r-project.org/web/packages/tibble/index.html
tidyr version 1.3.1	CRAN	https://cran.r-project.org/web/packages/tidyr/index.html
MACS2 version 2.2.9	Zhang *et al.*^23^	https://github.com/macs3-project/MACS
matrixStats version 1.3.0	CRAN	https://cran.r-project.org/web/packages/matrixStats/index.html
bamCoverage (deepTools version 3.5.5)	Ramírez *et al.*^24^	https://deeptools.readthedocs.io/en/latest/content/tools/bamCoverage.html
bamtofastq	10X Genomics	https://github.com/10XGenomics/bamtofastq
bedGraphToBigWig version 482	UCSC utilities	https://anaconda.org/channels/bioconda/packages/ucsc-bedgraphtobigwig/overview
bedToBigBed version 482	UCSC utilities	https://anaconda.org/channels/bioconda/packages/ucsc-bedtobigbed/overview
bedtools version 2.30.0	Quinlan *et al.*^25^	https://bedtools.readthedocs.io/en/latest/
bigWigToBedGraph version 482	UCSC utilities	https://anaconda.org/channels/bioconda/packages/ucsc-bigwigtobedgraph/overview
bowtie2 version 2.5.4	Langmead e*t al.*^26^	https://bowtie-bio.sourceforge.net/bowtie2/index.shtml
cellranger version 7.1.0	10X Genomics	https://www.10xgenomics.com/support/software/cell-ranger/latest/release-notes/cr-release-notes
perl version 5.32.1	Perl	https://www.perl.org/
picard version 2.23.8	RRID:SCR_006525	https://broadinstitute.github.io/picard/
samtools version 1.1.0	Danecek *et al.*^27^ Li *et al.*^28^	https://www.htslib.org/
scPASU pipeline	This paper	https://github.com/AngelaTingLab/scPASU_snakemake_version; https://doi.org/10.5281/zenodo.18342508
Snakemake version 7.32.4	Molder *et al*^29^	https://snakemake.github.io/
subset-bam version 1.1.0	10X Genomics	https://github.com/10XGenomics/subset-bam
umi_tools version 1.1.6	Smith *et al.*^30^	https://github.com/CGATOxford/UMI-tools

**Deposited data**		

Ureter scRNA-seq data	Fink *et al.*^31^	GEO: GSE184111
Urothelial cell gene and peak count matrices	Le *et al.*^1^	GEO: GSE262863

## Step-by-step method details

For the following sections, timing estimates are provided for the ureter dataset analyzed in Le et al.,^1^ with jobs running in parallel wherever possible. The Cell Ranger bam files for this dataset are approximately 384 GB; the pipeline timing will vary according to the initial dataset size and allocated HPC resources.

### Module 1: Data preprocessing

**Timing: 2–3 days, variable depending on dataset size and computational resources**

This module prepares the data for peak searching by removing PCR duplicates and ambiguous reads and performing genomic filtering using the polyAfilter package.

1. Submit Snakemake command to run module 1:

>snakemake run_module_1 --jobs [job_num] --cluster 'HPC_submit_parameters' --use-conda

***Note:*** This module contains nine core rules (Snakemake pipeline steps) that will execute when the run_module_1 command is run. *create_gtf_db* builds a reference database from the GTF file using polyAfilter and writes gtfb.db file to folder 1b. *preprocess_bam_dedup* and *preprocess_bam_cleanup* remove PCR duplicates and ambiguously mapped reads from Cell Ranger output bams, writing to folders 1a1 and 1a2 respectively. *genomicA_filtering_create_trans_file*, *genomicA_filtering_first_round*, and *genomicA_filtering_second_round* filter genomic-A primed reads (with and without intronic coverage) and write results to folder 1b. *subset_bams* subsets processed BAM files from folder 1b for cell types of interest using a user-provided list of cell barcodes, writing to folder 1c. *merge_bam_before_filt* and *merge_bam_after_filt* merge subsetted sample BAM files from folder 1c before and after genomic-A filtering respectively, writing to folder 1d.

***Note:*** This module contains two optional QC rules. *bw_track_before_filt* and *bw_track_after_filt* generate BigWig files for UCSC browser viewing.

***Note:*** If you are using Snakemake version 8.01 or greater, the --cluster command is deprecated. You will need to install the cluster-generic plugin and use that in the command line. Example command:

>snakemake run_module_1 --jobs [job_num] --executor cluster-generic --cluster-generic-submit-cmd 'HPC_submit_parameters' --use-conda

### Module 2: Peak calling and retention

**Timing: 24–30 h, variable depending on dataset size and computational resources**

This module calls candidate poly(A) site peaks from the processed reads and refines them using poly(A) junction evidence. Overlapping signals are split into distinct poly(A) sites, processing regions are delineated around supported sites, and unsupported artifacts are removed. To recover low-coverage sites, junction-only evidence is incorporated.

2. Submit Snakemake command to run module 2:

>snakemake run_module_2 --jobs [job_num] --cluster 'HPC_submit_parameters' --use-conda

***Note:*** This module contains nine rules. *all_filtered_reads_peak_call_summits* and *all_filtered_reads_sam_to_polyA* call read clusters with MACS2 and identify poly(A) junction reads with samToPolyA, outputting to all_filtered_reads (folder 2a) and after_genomicAfiltering (folder 2b). *polyAreads_sam_to_polyA* detects poly(A) junction reads, writing to before_genomicAfiltering (folder 2b). Then, *polyAreads_filterbam_byreadnames* and *polyAreads_peak_call_summits* subset the BAM file by these poly(A) junction reads and find clusters of poly(A) junction reads, outputting to before_genomicAfiltering under folder 2b and polyA_reads under folder 2a respectively. *bam_coverage_all_filtered_reads* and *bam_coverage_polyA_reads* identify alignment regions for each branch, writing to folder 2c1. *split_peak_ref_all_filtered_reads* and *split_peak_ref_polyA_reads* split multimodal signals if gaps exist, outputting to folder 2c2. *peak_ref_all_filtered_reads* and *peak_ref_polyA_reads* generate branch-specific poly(A) site peak reference files, retaining only junction-read supported peaks, outputting to folder 2d.

### Module 3: Peak cleanup and assignment

**Timing: 2–3 h, variable depending on dataset size and computational resources**

This module assigns candidate poly(A) site peaks to transcriptional units, applies stringent filters to remove artifacts and low-usage sites, and merges results from both processing branches into a dataset-specific peak reference. Peaks are then classified by genomic contexts, with fragmented sites flagged for caution.

3. Submit Snakemake command to run module 3:

>snakemake run_module_3 --jobs [job_num] --cluster 'HPC_submit_parameters' --use-conda

***Note:*** This module contains nine core rules. *create_turef* builds a transcriptional unit (TU) reference from the alignment GTF file, converting transcripts to TUs. Each TU is extended by 5kb in the 3′ direction and later updated to end at the most downstream peak within this flank region. *assign_tu_all_filtered_reads* and *assign_tu_polyA_reads* assign peaks that overlap with a single TU or its 3′ flank, outputting to folder 3a. *update_peak_ref_all_filtered_reads* and *update_peak_ref_polyA_reads* apply filters on processing-region length and minimum per-gene usage (set to 1,000 bp and 10% by default, respectively); optionally, they retain peaks based on poly(A) signal presence (PAS_filtering set to ‘true’) or proximity to annotated transcript end sites. These rules output to folders 3b1, 3b3, 3b4, and optionally 3b2. *merge_two_prongs* merges filtered peaks from both branches in folder 3b4 into a final peak reference, writing to folder 3c. *peak_classification* classifies peaks by genomic context, in priority order: 3′ UTR, TSS-proximal, Exonic, Intronic, or Flank. This rule outputs to folder 3d. *identify_fragmented_peaks* flags peaks likely fragmented by spliced alignments.

***Note:*** This module contains three optional QC rules. *make_filtered_tracks*, *plot_nuc_freq*, and *pa_ref_stats* generate BigWig tracks, a nucleotide frequency plot, and peak filtering statistics.

### Module 4: Poly(A) site peak-by-cell count matrix

**Timing: 3–4 h, variable depending on dataset size and computational resources**

This module uses the finalized poly(A) site reference to generate a per-sample peak-by-cell count matrix. It first converts the peak reference into a Cell Ranger-compatible reference, then converts the filtered BAMs back to FASTQ. Using these FASTQs and the peak reference, Cell Ranger produces the poly(A) peak-by-cell matrices for each sample. An optional step can also generate a standard gene-by-cell count matrix for downstream differential expression analysis.

4. Submit Snakemake command to run module 4:

>snakemake run_module_4 --jobs [job_num] --cluster 'HPC_submit_parameters' --use-conda

***Note:*** This module contains four rules. *cellranger_make_scPASU_ref* builds a Cell Ranger compatible reference from Module 3 peak reference. *bam_to_fastq* converts filtered BAM files from folder 1c to FASTQ, outputting to folder 4a. *cellranger_peakcount* uses FASTQs from folder 4a and peak reference to run cellranger count and write peak-by-cell count matrices to folder 4b. *cellranger_genecount* optionally generates gene-by-cell count matrices for downstream differential gene expression analysis.

### Module 5: Alternative poly(A) site usage testing and DEG analysis

**Timing: 30–60 min, variable depending on dataset size and computational resources**

This module provides optional R scripts designed to streamline common downstream analyses using the peak-by-cell count matrices generated in Module 4. It offers convenient workflows for merging count matrices, performing APA and differential gene expression testing, and generating browser-ready visualization tracks, but all steps are fully optional. Users may instead use the matrices directly for their own custom analyses.

5. Submit Snakemake command to run module 5:

>snakemake run_module_5 --jobs [job_num] --cluster 'HPC_submit_parameters' --use-conda

***Note:*** This module contains six rules. *merge_per_sample_peakcounts* and *merge_per_sample_genecounts* merge per-sample peak and gene count matrices and optionally edit cell barcodes to match with a user-provided Seurat object, outputting to folders 5a. *APA_testing* performs APA significance testing between two user-defined groups of cells to identify differentially expressed poly(A) sites, outputting to folder 5b. *DEG_testing* conducts pseudo-bulk differential gene expression (DEG) testing to generate a list of differentially expressed genes for comparison with APA peaks, outputting to folder 5b. *make_fractional_tracks* converts the APA bedGraph files generated by APA_testing to fractional usage track files for visualization in the UCSC Genome Browser. *make_track_annotations* generates a reference bigBed file annotating peaks for visualization in the UCSC Genome Browser.

## Expected outcomes

After completing Module 5, users can expect the “outputs” directory of the scPASU pipeline to be fully populated with all intermediate and final files generated throughout the workflow. Corresponding log files for each processing step are stored in the “logs” directory, providing useful information for troubleshooting in the event of pipeline errors. One of the key final products is the peak reference table, located in the “3e_fragmented_peaks_to_merge” directory and named “[file_prefix]_final_peak_universe_updated.txt.” This table contains the complete set of annotated poly(A) site peaks, formatted as [TU number]:[Gene name]:[Peak number], along with their genomic coordinates and genomic context classification. Peaks are numbered as P0 for genes with a single peak, or P1, P2, P3, and beyond for genes with multiple poly(A) sites, ordered from the most upstream to the most downstream. These annotations can be visualized in the UCSC Genome Browser using the bigBed files in the “track_annotations” directory, providing a clear overview of the refined poly(A) site landscape.

Users will also obtain merged gene-by-cell and peak-by-cell matrices across all samples, stored in the “5a_merged_cellranger_genecount” and “5a_merged_cellranger_peakcount” directories. These matrices represent key quantitative outputs and can be used directly for custom downstream analyses. APA-specific results are provided in the “5b_APA_testing” directory, which contains the “[group_1]_v_[group_2]_res.txt” summary file. This file reports pseudo-bulk sampling counts for each group, test statistics, and the “int_sig” column specifying whether each peak exhibits significant differential usage. Testing is performed using the DEXSeq package with three pseudo-replicates created by randomly sampling 70% of the cells within a testing group without replacement, and significant APA usage is defined by an adjusted p-value of less than 0.01, an absolute log2 fold change of greater than 1.5, and an absolute fractional usage difference of greater than 0.1. Fractional usage per peak is calculated by dividing the reads for each peak by the total number of reads across peaks for that gene, thus indicating the relative usage of each peak within a gene for each testing group. These fractional usage values for each cell group are included and can be visualized in the UCSC Genome Browser using the bigWig files in the “fractional_usage_tracks” directory.

**Figure 2:**
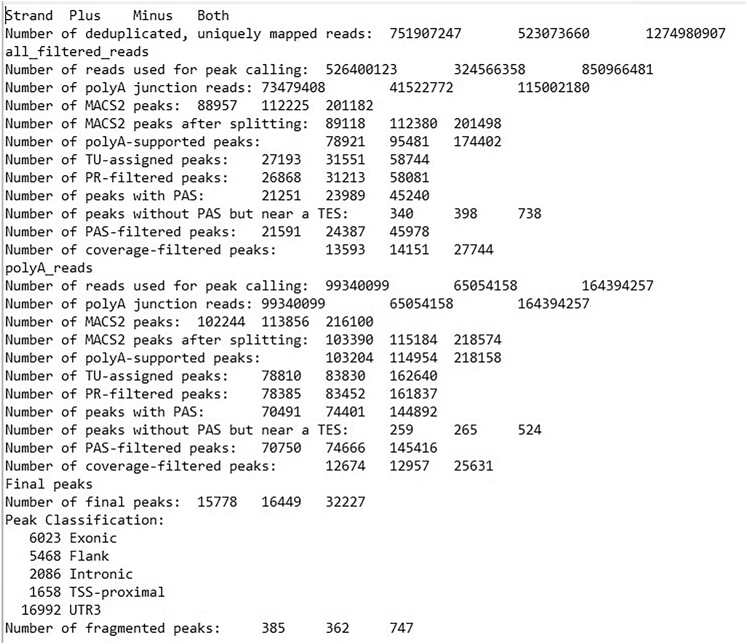
The PA_ref_stats file summarizes the read counts and peak counts recorded at each stage of the pipeline

Differential gene expression (DEG) outputs are provided in the “5b_DEG_testing” directory, including the “[group_1]_v_[group_2]_LRT_all_genes.txt” file containing results for all genes tested and the “[group_1]_v_[group_2]_LRT_sig_genes.txt” file listing significant DEGs. These outputs enable comparison between conventional transcriptional changes and APA-driven regulatory differences identified by the scPASU workflow. Additional quality-control information is provided in the “[file_prefix]_PA_ref_stats.txt” file, which summarizes read and peak counts at each filtering step (Figure 2).

**Figure 3:**
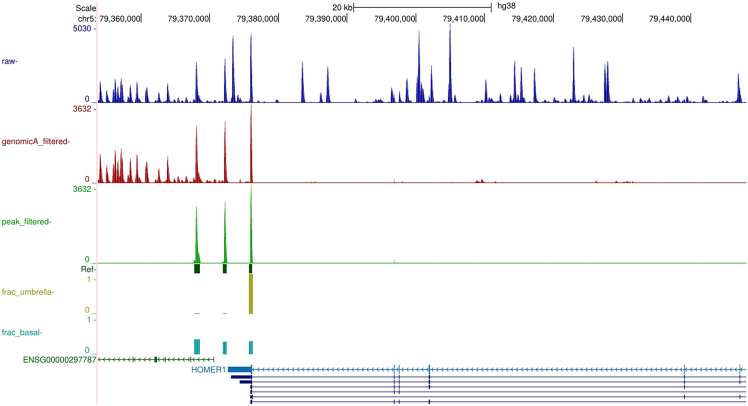
Example track visualization in the UCSC Genome Browser The top blue track represents the deduplicated, unambiguously mapped reads and corresponds to the “before_genomicAfiltering_merged_minus.bw file” in the “qc_ucsc_tracks” directory. The red track shows the reads remaining after genomic-A filtering and corresponds to the “after_genomicAfiltering_merged_minus.bw” file in the same directory. The green track displays the reads retained after all peak-level filtering steps in Module 3 and corresponds to the “[file_prefix]_peak_filtered_minus.bw” file in the “filtered_tracks” directory. The dark green annotated peaks correspond to the “[file_prefix]_peak_annotations_minus.bigBed” file in the “track_annotations” directory. The yellow and teal tracks represent the fractional usage values for each peak within a gene for the [group_one] and [group_two] cell types and correspond to the “[group_one]_v_[group_two]-[group_one]_minus.bw” and “[group_one]_v_[group_two]-[group_two]_minus.bw” files in the “fractional_usage_tracks” directory.

Several sets of bigWig tracks are also generated to visualize read retention across the major filtering steps of the workflow. The “qc_ucsc_tracks” directory contains stranded bigWig files from Module 1, including “before_genomicAfiltering_merged_minus.bw” and “before_genomicAfiltering_merged_plus.bw”, which show the reads remaining after removal of duplicated and ambiguous reads, as well as “after_genomicAfiltering_merged_minus.bw” and “after_genomicAfiltering_merged_plus_raw.bw”, which show the stranded read signal after the genomic-A filtering step. Final stranded read tracks are provided in the “filtered_tracks” directory as “[file_prefix]_peak_filtered_minus.bw” and “[file_prefix]_peak_filtered_plus.bw”, representing the fully filtered reads after all peak-level filtering steps in Module 3. When loaded into the UCSC Genome Browser (Figure 3), these progressive sets of tracks allow users to visually assess how each filtering step refines the data toward high-confidence poly(A) site peaks.

## Limitations

The scPASU pipeline has defined compatibility and computational requirements that may influence its applicability depending on the dataset and computing environment. At present, it is designed to operate with the mm10 and hg38 genome assemblies and therefore is intended for mouse and human analyses using these reference builds. The workflow also assumes BAM files produced using the Cell Ranger directory structure, which means that data generated with other single-cell alignment tools would require reformatting before use. As with most single-cell workflows, runtime and memory usage scale with the size of the input BAM files and the resources available on the computing system. Steps such as generating TRANS files for genomic-A filtering in Module 1 can be particularly resource-intensive and may run more slowly on systems with limited RAM. These factors do not affect the validity of the results but may influence execution speed or feasibility depending on the user’s infrastructure and dataset characteristics.

## Troubleshooting

### Problem 1

The *preprocess_bam_dedup* rule fails with a MissingInputException error. Related to module 1: data preprocessing.

### Potential solution

This error typically indicates that the pipeline cannot find the expected Cell Ranger–generated BAM files. The rule looks for each BAM at [cellrangeroutputpath]/[sample]/outs/possorted_genome_bam.bam. Begin by opening the “config.yaml” file and confirming that cellrangeroutputpath points to the correct directory and that it is specified as an absolute path. Next, verify that the entries under samples match the Cell Ranger output folder names exactly. Finally, ensure that the BAMs were produced with cellranger count, which yields the directory structure the workflow expects; outputs from cellranger multi have a different layout and will not be detected unless reformatted to match the standard count structure.

### Problem 2

A *split_peak_ref* step fails, and the log file contains the message: “Error in library(goldmine, lib.loc = libpath): there is no package called goldmine”. Related to module 2: peak calling and retention.

### Potential solution

This message indicates that the *goldmine* package is not being recognized by R during the execution of the workflow. To confirm that the package is installed correctly, open an R session outside the pipeline, navigate to the directory where the package is located, and attempt to load it by running library(goldmine). If the package fails to load, reinstalling it should resolve the issue. If the package loads successfully in a standalone R session, review the goldminepath entry in the “config.yaml” file to ensure it is formatted correctly. The path should be an absolute path pointing to the directory that contains the installed *goldmine* package; for example, if it is installed within an R library, the variable should be set to a directory of the form “/path/to/your/R_lib_path/library”.

### Problem 3

Many robust-looking peaks in the raw and genomic-A filtered tracks are missing from the peak-filtered tracks. Related to module 3: peak clean up and assignment.

### Potential solution

This outcome generally indicates that these peaks were removed during the filtering steps in Modules 2 or 3. A common reason for exclusion is insufficient support from poly(A) junction reads. The default requirement is a minimum of three supporting junction reads, but datasets with low sequencing depth may naturally fall below this threshold. In such cases, lowering the cutoff to two or even one read may be appropriate, depending on the characteristics of the dataset. Another parameter that can influence peak retention is the optional poly(A) signal (PAS) filtering step, which keeps peaks only if they contain or are near a canonical poly(A) hexamer motif or fall within a defined distance of an annotated transcription end site. While this filter improves specificity, it may remove legitimate peaks in certain contexts; for example, in cancer samples where mutations can disrupt hexamer motifs. If peaks appear to be lost due to this criterion, users may disable this step by setting the PAS_filtering parameter to “false” in the “config.yaml” file.

### Problem 4

The APA testing or DEG testing rules fail during execution. Related to module 5: alternative poly(A) site usage testing and DEG analysis.

### Potential solution

These failures are often caused by mismatches between the cell barcodes in the metadata file and those in the peak by cell matrix located in the “5a_merged_cellranger_peakcount” directory. Even small differences in formatting, such as variations in barcode suffixes, can prevent the testing steps from running, especially when the metadata originates from a separate scRNA-seq analysis that uses a different barcode convention. To resolve this issue, check that the barcodes match exactly between the metadata file and the matrices produced by the pipeline. Also verify that the values specified for group_one, group_two, and meta_testing_column in the “config.yaml” file correspond precisely to the labels used in your metadata. Ensuring consistency across these fields will allow the APA and DEG testing rules to run successfully.

### Problem 5

When viewing the UCSC Genome Browser tracks, many peaks annotated in the reference bigBed file do not have corresponding bars in the fractional usage tracks. Related to module 5: alternative poly(A) site usage testing and DEG analysis.

### Potential solution

This is expected for all peaks labeled P0. These peaks represent genes that contain only a single poly(A) site, and because differential APA analysis requires at least two sites within a gene, P0 peaks are not included in fractional usage calculations. Peaks may also be absent from the fractional usage tracks if they are expressed in fewer cells than the minimum percentage required for APA testing. This threshold is controlled by the min_cell_expr_pct parameter, which is set to 10 by default. For datasets with sparse coverage or rare cell populations, adjusting this value in the “config.yaml” file may allow more peaks to be included in the analysis.

## Resource availability

### Lead contact

Further information and requests for resources and reagents should be directed to and will be fulfilled by the lead contact, Angela H. Ting (ahting@mdanderson.org).

### Technical contact

Technical questions on executing this protocol should be directed to and will be answered by the technical contact, Alexandra Krylova (aekrylova@mdanderson.org).

### Materials availability

This study did not generate new unique reagents.

### Data and code availability

Ureter scRNA-seq data are available at GEO under accession number GEO: GSE184111. The gene and peak count matrices are available under GEO: GSE262863. Codes are available at https://github.com/AngelaTingLab/scPASU_snakemake_version. A version of record has been archived at Zenodo under the DOI: https://doi.org/10.5281/zenodo.18342508.

## Acknowledgments

This work was supported by the 10.13039/100000062National Institute of Diabetes and Digestive and Kidney Diseases (NIDDK) under grant U01DK131383 to A.H.T. The authors also acknowledge the High Performance Computing for Research facility at The University of Texas MD Anderson Cancer Center for providing the computational resources that contributed to the results reported in this study.

## Author contributions

Conceptualization, N.B.L. and A.H.T.; methodology, A.K. and N.B.L.; investigation, A.K. and N.B.L.; visualization, A.K.; funding acquisition, A.H.T.; project administration, A.H.T.; supervision, A.H.T.; writing – original draft, A.K.; writing – review and editing, N.B.L. and A.H.T.

## Declaration of interests

The authors declare no competing interests.
